# Myricetin Suppresses Ovarian Cancer *In Vitro* by Activating the p38/Sapla Signaling Pathway and Suppressing Intracellular Oxidative Stress

**DOI:** 10.3389/fonc.2022.903394

**Published:** 2022-05-11

**Authors:** Qi Li, Qi Tan, Yangfei Ma, Zehui Gu, Suxian Chen

**Affiliations:** Department of Pathology, The Third Affiliated Hospital of Jinzhou Medical University, Jinzhou, China

**Keywords:** ovarian cancer, Myricetin, oxidative stress, proliferation, apoptosis

## Abstract

Ovarian cancer is a common malignancy with a mortality and effective, efficient treatments are urgently needed. Myricetin (Myr) is a flavonoid with antioxidant and anticancer properties. Here, we assessed Myr’s toxicity on the non-tumor cell line, IOSE-80 and the mechanism by which it suppresses proliferation, migration, and invasion of ovarian cancer SKOV3 cells. The effects of Myr on SKOV3 cells were assessed using CCK-8, oxidative stress, wound healing, Transwell, Hoechst 33258 staining, and western blot assays. Our data show that although Myr was not toxic against IOSE-80 cells for a range of concentrations 0-40μM, it suppressed SKOV3 cell proliferation, migration, and invasion and enhanced apoptosis. Mechanistically, it activated the p38/Sapla signaling pathway, thereby inhibiting oxidative stress and reducing the level of ROS in tumor cells. Our data show that Myr suppresses ovarian cancer cells *in vitro* and suggests Myr as a candidate agent against ovarian cancer.

## Introduction

Cancer is one of the top life-threatening dangers to the human survival, accounting for over 10 million deaths per year (1). Ovarian cancer (OC), which affects about 225,000 women annually (2), is the 3^rd^ commonest gynecological malignancy worldwide and is associated with a high mortality rate, killing about 125,000 people annually (2, 3). Current OC treatments include surgery, radiotherapy, chemotherapy, and a combination of the two. Despite advances in OC treatment strategies, its 5-year survival rate is about 40% (4). Moreover, almost all cancer chemotherapies cause significant toxicity to normal cells. Thus, there is an urgent need for new therapeutics with higher efficiency and fewer side effects.

Myricetin (3, 5, 7-trihydroxy-2-(3,4,5-trihydroxyphenyl)-4-chromenone, Myr), is a phenolic compound found in different plant-based dietary agents, including fresh fruits, vegetables, and herbs. It has nutraceutical effects and antioxidant properties. Its pharmacological activities include anti-inflammatory, antioxidant, antibacterial, antiviral, liver protection, and cardioprotective effects (4–6). Recent studies show that Myr has anti-cancer cells activity and markedly low toxicity to normal cells. Myr is reported to enhance chemotherapy sensitivity in OC cell lines that are resistant to chemotherapeutics like paclitaxel (PTX) and doxorubicin (DOX) (7). Due to its low toxicity to normal cells, Myr has attracted attention as a potential anti-cancer agent (8–10). Myr inhibits cancer by inducing apoptosis, cell cycle arrest, nanobiovectors, immunotherapy, and antagonizing drug resistance in tumor cells (11).

The mitogen-activated protein kinase (MAPK) family regulates oxidative stress and pathophysiological processes in cells. This family comprises many subfamilies. Among them, p38 is the most important member that is involved in the regulation of various biological processes in mammalian cells, such as cell growth, differentiation, apoptosis and oxidative stress. Originally described as a tumor-suppressor kinase that inhibits RAS-dependent transformation, p38 has also been shown to function as a tumor promoter. On this basis, several inhibitors targeting p38 have been designed and tested in clinical trials for the treatment of several human malignancies, although without much success to date (12). Elucidating various aspects of p38 functions might uncover new possibilities for developing more effective p38-based therapies. The Saplap protein acts downstream of p38 and is a protein phosphatase regulatory subunit. In this study, we investigated the effect of myricetin on ovarian cancer cells, and whether its effects were mediated by p38/Sapla signaling pathway. The results of this study provide a theoretical basis for the development of myricetin-based drugs for cancer treatment.

## Materials and Methods

### Reagents and Materials

Myr was purchased from Jingzhu Pharmaceutical Co., Ltd. (529-44-2). The CCK-8 assay kit (96992-100TESTS-F) was purchased from Sigma. The BCA assay kit (P0011) and Hoechst 33258 (C1017) were purchased from Beyotime Institute of Biotechnology. Transwell chambers (3422, BD), artificial basement membrane (356234, BD), 0.5% crystal violet (60506ES60), and reactive oxygen species assay kit (50101ES01) were purchased from Yeasen Biotech Co., Ltd. LDH (lactate dehydrogenase, A020-2), MDA (malondialdehyde, A003-1) and SOD (superoxide dismutase, A001-3) detection kits were purchased from Nanjing Jiancheng Institute of Biotechnology.

### Cell Culture

IOSE-80 (CP-H055), a non-tumor cell line and SKOV3 (CL-0215), an OC cell line, were purchased from Procell Life Science & Technology Co. Ltd and cultured in RPMI 1640 (SH30243.01, Hyclone) supplemented with 10% FBS (SH30084.03, Hyclone) and 1% pen/strep (P1400, Solarbio) at 37°C in a humidified incubator, 5% CO_2_.

### CCK-8 Viability Assays Analysis

Working solutions of Myr (1×10^-7^, 1×10^-6^, 1×10^-5^ and 1×10^-4^M) were prepared in serum-free RPMI 1640 media. SKOV-3 cells were seeded in 96-well plates at 5×10^4^ cells/well and cultured for 6, 12, 24, and 36 h. Next, 10μL of CCK8 were added into each well and cells incubated at 37°C for 2 h. Absorbance (A) was then read at 450nm on a microplate analyzer. Cell viability was calculated using the formula: experimental group A value/control group A value ×100%. Based on this analysis, an optimal Myr concentration range of 10-50μM, and treatment duration of 24 h were selected for subsequent experiments. Next, IOSE-80 and SKOV3 cells were treated with Myr at 0, 10, 20 and 40 μM for 24 h and subjected to CCK8 viability assays as described in section 2.3. The experiment was repeated for three times independently.

### Analysis of SKOV3 Cell Morphology

Cells were seeded in 6-well plates at 1×10^6^ cells/well and cultured for 24 h. They were then treated with different concentrations of Myr and divided into control group (0μM Myr) and experimental group (10, 20 and 40 μM Myr) for 24 h, followed by morphological examination imaging under an inverted microscope.

### Detection of ROS Levels in SKOV3 Cells

A ROS detection kit was used to determine intracellular ROS levels in each treatment group. Briefly, cells were seeded in 6-well plates at 1×10^6^ cells/well and cultured for 24 h before treatment with Myr for 24 h. Media was then replaced with 1mL of 10μM DCFH-DA and the cells incubated at 37°C for 30 min, followed by imaging on a fluorescence microscope. The experiment was repeated for three times independently.

### Detection of MDA, SOD, and LDH Levels in SKOV3 Cells

Cells were seeded in 6-well plates at 1×10^6^ cells/well and allowed to adhere. They were then treated with Myr for 24 h. The culture medium was collected for measurement of MDA, SOD, and LDH levels using the thiobarbituric acid method, xanthine oxidase method, and dinitrophenylhydrazine colorimetric method, respectively, following instructions on respective kits. The experiment was repeated for three times independently.

### Cell Migration Assay

A wound-healing assay was carried out to explore the migration of SKOV3 cells. Cells were grown to 80-90% confluence in 24-well plates, and a wound created by scratching across the monolayer with a plastic pipette tip. Remaining cells were then washed thrice with PBS to clear cellular debris and incubated at 37°C in serum-free media. Migrating cells at the wound front were photographed after 24 h. The experiment was repeated for three times independently.

### Transwell Migration and Invasion Assay

Cell migration assays were done on non-Matrigel coated 24-well Transwells (8μm pore size, Corning). Cell invasion assays were done on Matrigel pre-coated 24-well Transwells (8 μm pore size, Corning). 1×10^5^ cells in 500μL RPMI 1640 supplemented with 1% FBS were added into the upper chamber and 750μL RPMI 1640 containing 10% FBS added into the lower chamber. After 24 h incubation, the Matrigel and the cells remaining in the upper chamber were removed using cotton swabs. Cells on the lower surface of the membrane were then fixed with 4% PFA and stained with 0.5% crystal violet. Cells in 5 fields of view were then counted and imaged at ×100 magnification. The experiment was repeated for three times independently.

### SKOV3 Apoptosis Detection Using Hoechst 33258

Cells were seeded on 6-well plates at 1×10^6^ cells/well and allowed to adhere. They were then treated with Myr for 24 h. Subsequently, the cells were collected and washed thrice with pre-cooling phosphate-buffered saline (PBS). They were then fixed with 0.5mL 10% formaldehyde at 4°C for 10 min and stained with 0.5mL of Hoechst 33258 at room temperature for 3 min. Apoptosis morphological changes were then examined and imaged by fluorescence microscopy. The experiment was repeated for three times independently.

### Western Blot Analysis

Cells were lysed using RIPA buffer and proteins resolved by SDS-PAGE. They were then transferred onto PVDF membranes (Millipore, USA) and blocked with 5% milk, followed by incubation with primary antibodies at 4°C overnight. The membranes were washed and incubated with HRP-conjugated secondary antibodies for 2 h at room temperature. The following antibodies were purchased from ABclonal Technology Co., Ltd (Wuhan, China): p38 (A14401, 1:1000), p-p38 (A17080, 1:800), Sapla (PPP6R3, A17177, 1:800), EGFR (A4929, 1:1000), MMP-2 (A19080, 1:1000), MMP-3(A11418, 1:1000), MMP-9 (A0289, 1:1000), Bax (A19684, 1:1000), Bcl-2 (A19693, 1:800), active + pro caspase-3 (A19654, 1:1000), caspase-9 (A18676, 1:1000), GAPDH(A19056, 1:1000), HRP Goat Anti-Rabbit IgG (H+L) (AS014, 1:5000). The experiment was repeated for three times independently.

### Data Analysis

All results are presented as mean ± standard deviation (SD). Data analysis was performed with the SPSS 19.0 and GraphPad 8.0 software. The student’s *t*-test or one-way ANOVA was applied to compare data from different groups. Statistical significance was defined as p < 0.05.

## Results

### Myricetin Had No Toxic Effect on IOSE-80 Cells and Inhibited SKOV3 Cell Proliferation

**Figure 1:**
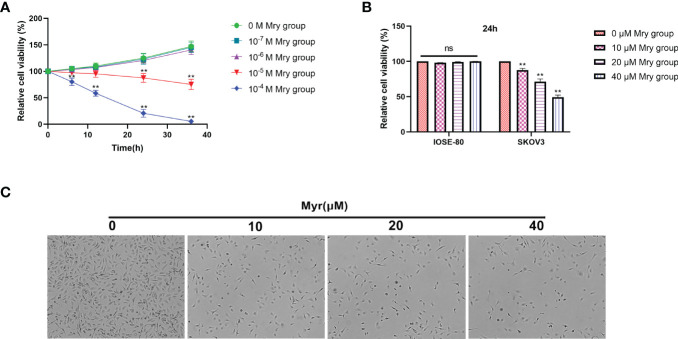
Effects of different concentrations of myricetin on cell cytotoxicity/viability. Determination of cell viability using CCK-8 assay and Myr cytotoxicity at 0, 10, 20 and 40μM. **(A)** The effect of Myr on the viability of SKOV3 cells at different doses and times were determined by CCK-8 analysis. **(B)** Cytotoxic effect of different Myr concentrations on IOSE-80 cells and SKOV3 cells was determined by CCK-8 analysis. **(C)** SKOV3 cells were incubated with 0, 10, 20 and 40μM Myr for 24 h. Representative microscopic images are shown (×40 magnification). n = 3, **p<0.01 vs. control.

CCK-8 cell viability/cytotoxicity assays showed that the optimal concentration range for Myr inhibition of SKOV3 proliferation was 1×10^-5^-1×10^-4^ M for 24 h (**Figure 1A**). Of note, the viability of cells treated with Myr at concentrations of 1×10^-4^M for 24 h was too low. Therefore, low concentrations (0-50 μM) were chosen for subsequent experiments. At these concentrations, Myr was not toxic to IOSE-80, non-tumor cells but was inhibitory on SKOV-3 cells in a dose-dependent manner (**Figure 1B**). SKOV3 cells treated with Myr appeared smaller, with a reduced cell, and high rate of cell death (**Figure 1C**).

### Myr Inhibits Oxidative Stress in SKOV3 Cells

**Figure 2:**
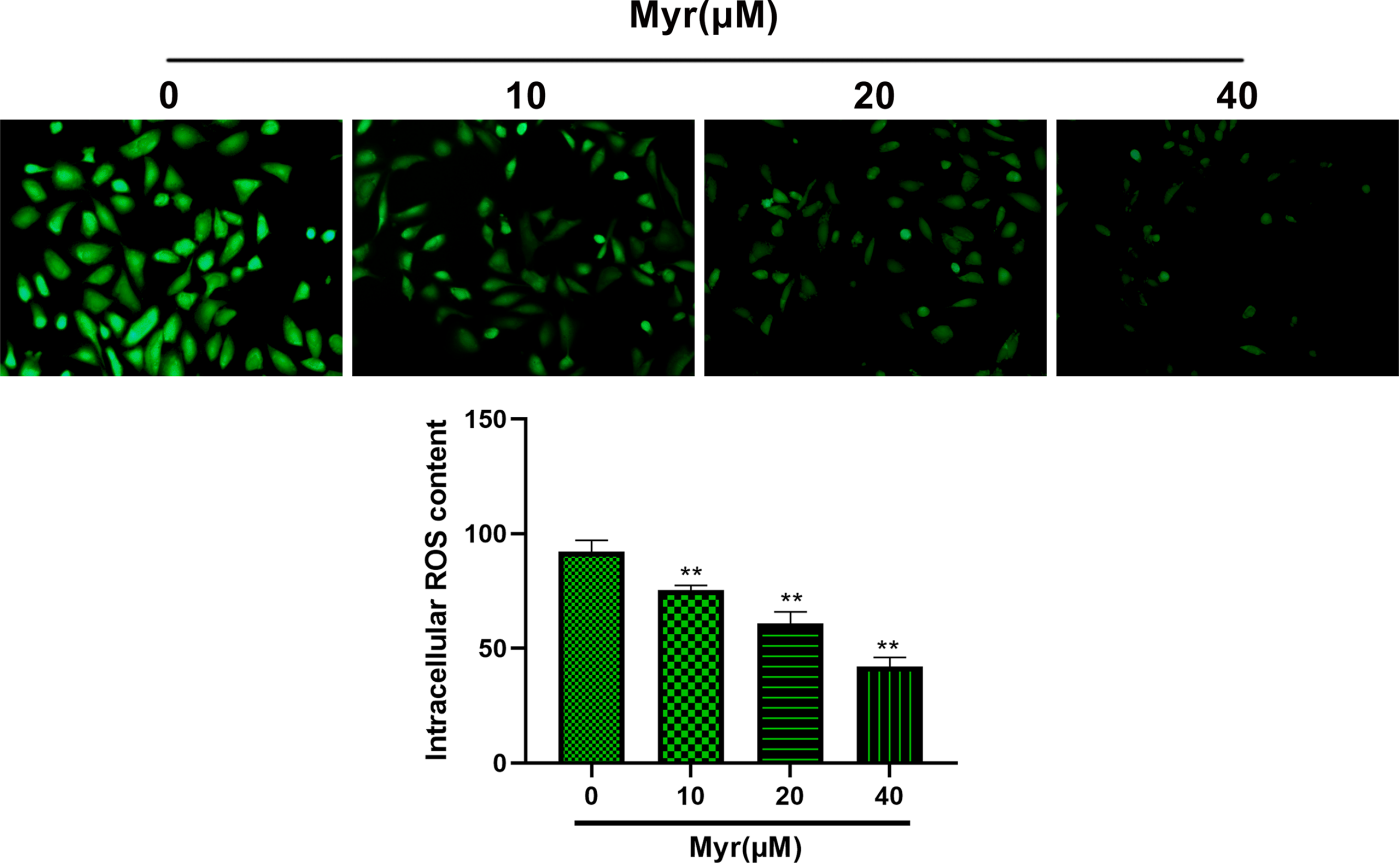
Effects of different concentrations of myricetin on ROS levels in SKOV3 cells. After treating SKOV3 cells with Myr at 0, 10, 20 and 40 μM for 24 h, ROS assay was used to measure intracellular ROS levels. Representative fluorescence images are shown (×200 magnification). n=3; **p<0.01 vs. control.

Relative to controls, ROS levels in SKOV3 cells were significantly reduced by Myr at 10, 20 and 40 μM in a dose dependent manner (**Figure 2**, *p*<0.01). Relative to controls, intracellular MDA was significantly reduced dose-dependently by Myr at 10, 20 and 40 μM while SOD levels increased significantly. LDH levels in the culture medium also reduced significantly (**Table 1**, *p*<0.05 or *p*<0.01).

**Table 1:** Effects of different concentrations of Myr on MDA, SOD and LDH in SKOV3 cells.

Group	MDA (nmol·mL^-1^)	SOD (U·L^-1^)	LDH (U·L^-1^)
0 μM Myr	6.13 ± 2.45	9.50 ± 3.62	80.78 ± 6.03
10 μM Myr	5.58 ± 0.72*	14.11 ± 2.33**	72.26 ± 4.81*
20 μM Myr	4.11 ± 1.06**	20.62 ± 2.17**	63.03 ± 5.15**
40 μM Myr	2.26 ± 0.31**	29.90 ± 3.25**	51.49 ± 3.03**

Intracellular MDA levels were evaluated using the MDA assay. Intracellular SOD activities were evaluated using the SOD assay. MDA levels in culture media were evaluated using the LDH assay. Data are expressed as mean ± SD. n=3; *p<0.05, **p<0.01 vs. control.

### Myr Inhibits the Migration and Invasion of SKOV3 Cells

**Figure 3:**
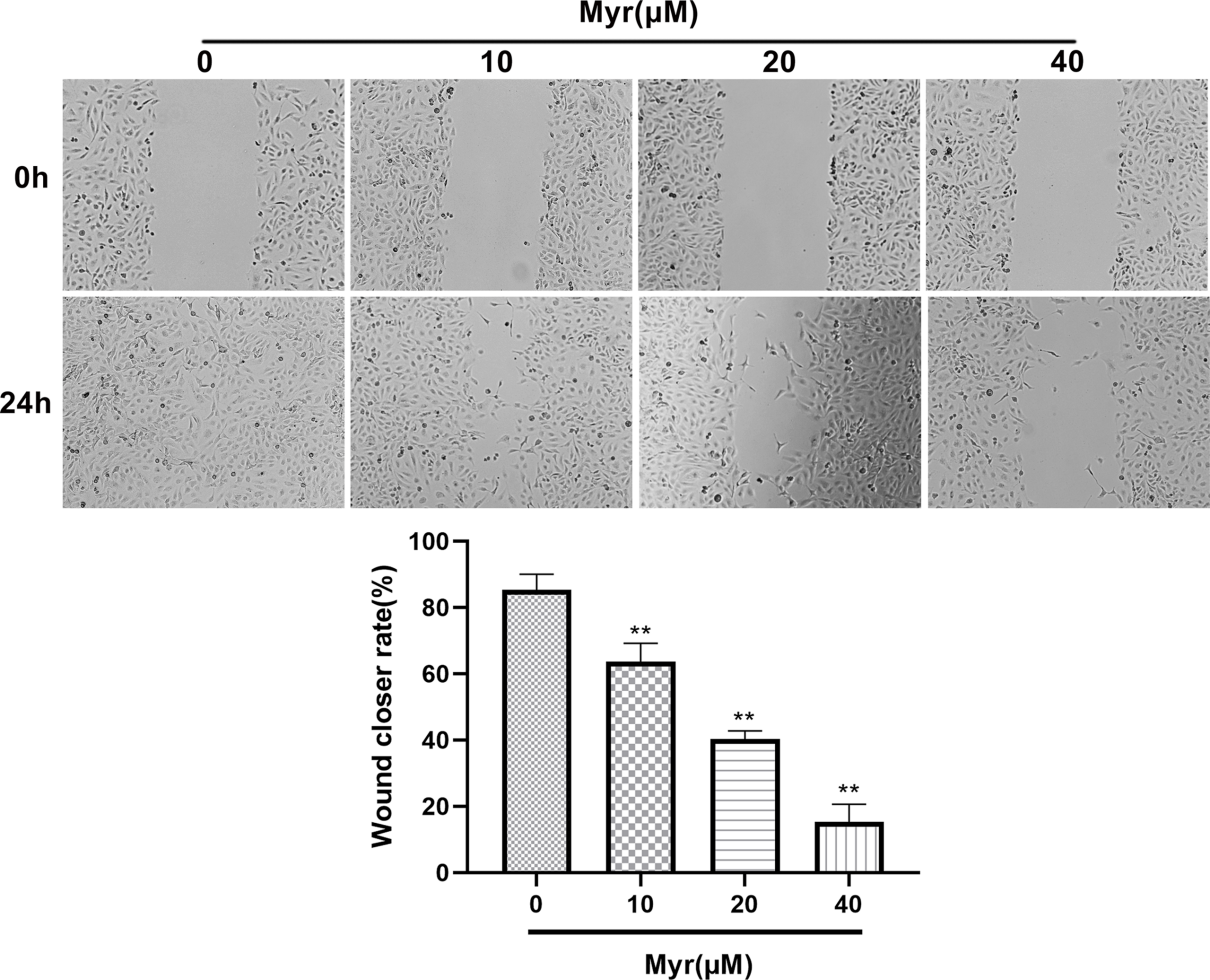
Effects of different concentrations of Myr in SKOV3 wound healing. After treating SKOV3 cells with Myr at 0, 10, 20 and 40 μM for 24 h, cell migration was analyzed using a wound healing assay and microscopy at ×40 magnification. n=3; **p<0.01 vs. control.

**Figure 4:**
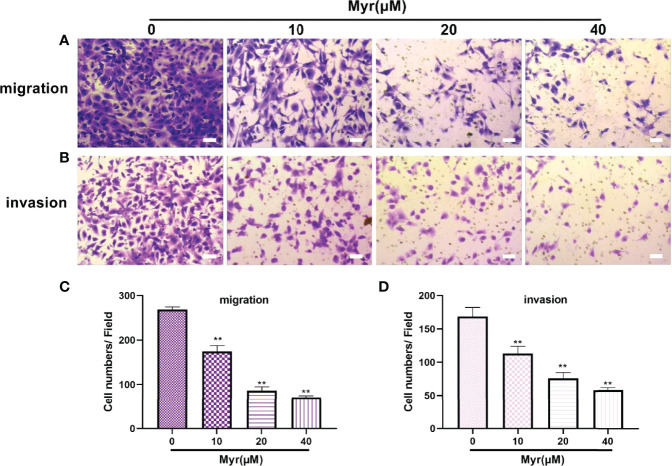
Effects of different concentrations of Myr on SKOV3 migration and invasion. After treating SKOV3 cells with Myr at 0, 10, 20 and 40 μM for 24 h, migration **(A, C)** and invasion **(B, D)** were determined using Transwell assays and microscopy at ×100 magnification. n=3; **p < 0.01 vs. control.

Wound-healing and Transwell analyses of whether Myr inhibits migration and invasion by SKOV3 cells, revealed that relative to controls, Myr (10, 20 and 40 μM) slowed wound closure dose-dependently (*p*<0.01, **Figure 3**). Relative to controls, treatment with Myr significantly reduced the number of SKOV3 cells migrating downward (*p*<0.01, **Figures 4A**–**C**) and invading Matrigel (*p*<0.01, **Figures 4B**–**D**) in a dose-dependent manner.

### Myr Promoted Apoptosis in SKOV3 Cells

**Figure 5:**
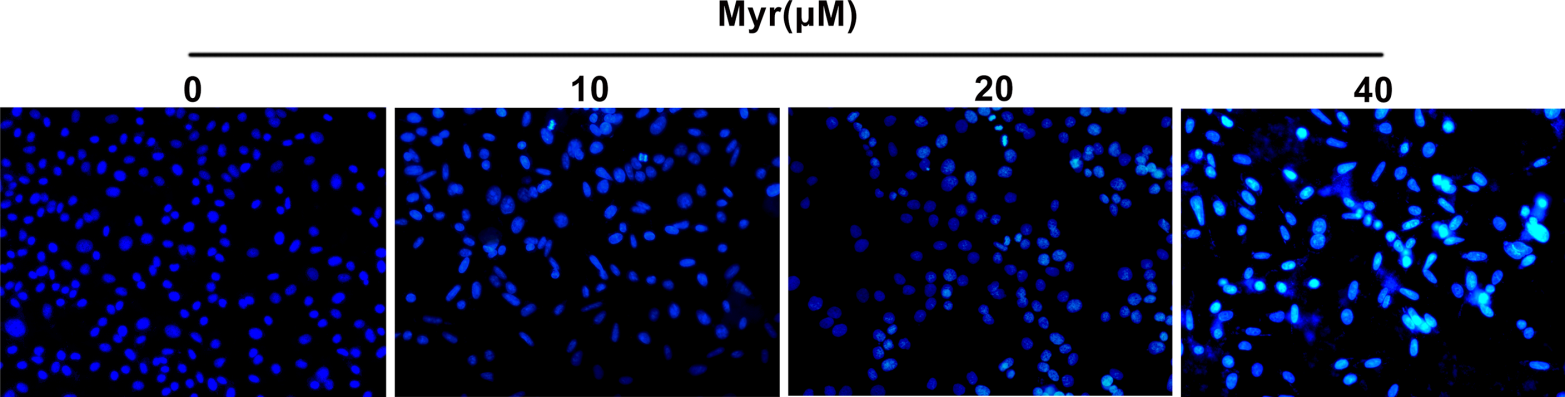
Effects of different concentrations of Myr on SKOV3 apoptosis. After treating SKOV3 cells with Myr at 0, 10, 20 and 40 μM for 24 h, Hoechst 33258 was used to detect apoptosis. Representative fluorescence images are shown at ×100 magnification. n=3.

Relative to control SKOV3 cells, Hoechst 33258 staining revealed that the nuclei of cells treated with various Myr concentrations were densely stained or fragmented, with visible apoptotic bodies and the apoptotic effect was dose-dependent (**Figure 5**).

### Myr Affected the Expression of Proliferation-, Migration-, and Invasion-Related Proteins in SKOV3 Cells

**Figure 6:**
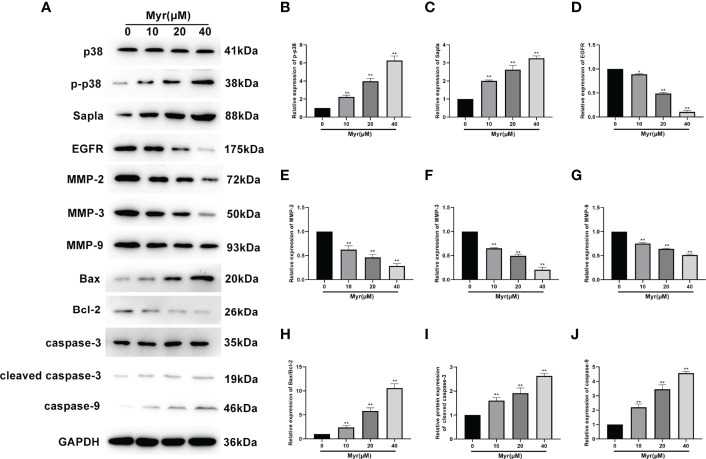
Effects of different Myr concentrations on expression of proliferation-, migration-, and invasion-related proteins in SKOV3 cells. **(A)** Western blot analysis image. Relative levels of **(B)** p-p38, **(C)** SAPLA, **(D)** EGFR, **(E)** MMP2, **(F)** MMP3, **(G)** MMP9, **(H)** Bax/Bcl-2, **(I)** cleaved caspase-3, and **(J)** caspase-9, were determined using western blot analysis. n=3; *p<0.05, **p<0.01 vs. control.

Next western blot analysis was used to determine the levels of the proliferation-related proteins, p38, p-p38, SAPLA, and EGFR), the migration- and invasion-related proteins, MMP2, MMP3, and MMP9, and the apoptosis-related proteins, Bax, Bcl2, cleaved caspase-3 and caspase-9 in SKOV3 cells. These analyses revealed that relative to controls, the levels of p-p38, SAPLA, Bax/Bcl-2, cleaved caspase-3 and caspase-9 were significantly increased in SKOV3 cells treated with various Myr concentrations (*p*<0.01, **Figure 6**) while the levels of EGFR, MMP2, MMP3 and MMP9 decreased significantly (*p*<0.05 or *p*<0.01). These effects on protein expression were dose-dependent.

## Discussion

The quality of life of ovarian cancer (OC) patients is poor, with about 75% of the patients experiencing recurrence within 2 years. This is attributable to chemotherapy’s toxicity and OC resistance to chemotherapeutics (13). Thus, effective anti-OC drugs with less toxicity and fewer side effects are urgently needed. Recent studies have proposed edible flavonoids as potential anticancer candidates. Epidemiological studies show that eating fruits, vegetables, and whole grains containing phytochemicals like flavonoids reduces cancer risk. Eating more fruits, vegetables, and fish containing flavonoids is reported to reduce breast cancer risk (14). Given that flavonoids may have better anticancer effects than conventional chemotherapeutics, we investigated the effects of myricetin (Myr) against OC aiming to elucidate the underlying mechanisms of its anticancer effect.

Myr was originally isolated from the bark of the tree Myrica rubra (Myrica rubra (Lour.) S. et Zucc.). Its chemical formula is C_15_H_10_O_8_ and the relative molecular mass is 318.24. According to reports, Myr inhibits the proliferation of various cancer cells through multiple signaling pathways, including PI3K/AKT, PAK1/MEK/ERK1/2, cyclin D1/PCNA, STAT3 and so on (15, 16). A study found that, Myr is able to interact with the human telomere G-quadruplex TTAGGG 3 DNA in human breast cancer MCF-7 cells, thus inhibiting human telomerase reverse transcriptase mRNA and telomerase in a concentration-dependent manner, thereby inhibiting cell proliferation (17). Bitew et al. showed that Myr are not hepatotoxic and cytotoxic (18). Here, we found that at 10, 20 and 40 μM, Myr significantly reduces the viability of SKOV3, an OC cell line but not that of IOSE-80 cells, a non-tumor cell line, indicating that Myr can inhibit the proliferation of OC cells without toxicity on non-tumor cells. This is consistent with previous reports that Myr’s inhibitory effects on cells are selective (19, 20), highlighting its potential as a safe anti-OC agent.

Reactive species, which mainly include reactive oxygen species (ROS), are byproducts of metabolic reactions in mitochondria. In normal cells, low-level concentrations of these compounds are required for signal transduction before their elimination. However, cancer cells, which have higher metabolism, require high ROS levels to maintain high proliferation rates. The implications for ROS regulation are highly significant for cancer therapy because commonly used radio- and chemo-therapeutic drugs influence tumor outcome through ROS modulation. Oxidative stress is a chemical reaction that generates free radicals through different chain reactions. Increased levels of free radicals or ROS in cells are a signs of cancer. Studies have found that ROS levels in tumor cells are generally high (15). Importantly, Myr has antioxidant effects and can eliminate ROS. Antioxidants defend against high levels of reactive species (especially ROS) (21) and there is evidence that they prevent tumorigenesis and extend life span (22). Apart from their protective roles against cancer, antioxidant supplementation during chemotherapy may lower dose-limiting toxicities (23). Here, we found that Myr effectively reduces ROS levels in SKOV3 cells, dose-dependently. MDA is an indicator of ROS and SOD catalyzes the reduction of superoxide anion radicals to hydrogen peroxide. 3,4-Methylenedioxyamphetamine often interacts with SOD to indirectly reflect the severity of free radical attack and ability of oxygen free radical scavenging. On the other hand, LDH is a metalloprotein containing zinc ions and is an important enzyme involved in anaerobic glycolysis and gluconeogenesis. Elevated LDH is common in diseases like myocardial infarction, skeletal muscle injury, and cancer (24). Here, Myr significantly reduced MDA and LDH levels in SKOV3 cells, and dose-dependently increased SOD activity. Our data show that Myr inhibits oxidative stress in SKOV3 cells, reduces ROS production, and enhances scavenging of oxygen free radicals.

Oxidative stress affects cell proliferation *via* various signaling pathways, including EGFR and mTOR pathways that involve key signaling proteins like Ras, Raf, and MAPK pathway factors like ERK1/2, MEK, and p38 (25, 26). p38 is a key sensor for oxidative stress, and its redox-sensing function is essential for controlling tumor development (27). In contrast to other MAPKs, p38 suppresses tumorigenesis by blocking proliferation or promoting apoptosis. Our data show that Myr activates p38 expression in SKOV-3 cells, enhances p-p38 and Salpa expression, suppresses EGFR expression, and inhibits cell proliferation.

Epithelial-mesenchymal transition (EMT) is an important driver of metastasis. Numerous ROS-related signaling pathways are involved in EMT, including matrix metalloproteinases (MMPs). The impact of ROS as an EMT mediator is also supported by MMP3 (matrix metalloproteinase 3), which catalyzes extracellular matrix breakdown, thereby driving metastases by degrading collagen, fibronectin, and laminin. MMP3 is reported to be upregulated in cancer, including breast cancer and is an EMT inducer in transgenic mice. In mice, MMP3 secretion is associated with Snail upregulation, E-cadherin loss, and catenin nuclear translocation, which, in turn, are dependent on the small GTP-binding protein Rac1 (Rac1b) (28). Other metalloproteinases, including MMP2 and MMP9, play roles in Rac1b stimulation to influence ROS. Most of these proteins are oncogenic in mouse models, and their overexpression contributes to human tumorigenesis. Here, wound healing and Transwell assays revealed that Myr inhibits SKOV3 cells invasion and metastasis. Western blot analysis showed that Myr significantly inhibited the expression of MMP-2, MMP-3 and MMP-9, suggesting that Myr can prevent oxidative stress in SKOV3 cells, thereby suppress cell invasion and metastasis. Additionally, the correlation between decreased ROS generation and caspase-9 activity, and increased caspase-3 activity confirmed Myr’s antioxidant and proapoptotic activity in SKOV-3 cells. Hoechst 33258 staining revealed Myr-induced apoptosis in SKOV3 cells. Moreover, treatment with Myr significantly promoted the expression of apoptosis-related proteins (Bax/Bcl-2, cleaved caspase-3 and caspase-9). These results were in line with those of cell wound healing, transwell, and Hoechst 33258 staining assays, suggesting that Myr prevents migration and infiltration, as well as enhances apoptosis of SKOV3 cells.

In summary, Myr has an anti-ovarian cancer effect *in vitro*, and the mechanisms underlying this may be related to oxidative stress in tumor cells. By inhibiting intracellular ROS production, Myr may inhibit proliferation, migration, and invasion in SKOV3 cells, thereby enhancing apoptosis. Our data highlight Myr as a promising candidate for OC prevention and adjuvant therapy.

## Data Availability Statement

The original contributions presented in the study are included in the article/**Supplementary Material**. Further inquiries can be directed to the corresponding author.

## Ethics Statement

The studies involving human participants were reviewed and approved by The Institutional Research Ethics Committee of The Third Affiliated Hospital of Jinzhou Medical University. The patients/participants provided their written informed consent to participate in this study.

## Author Contributions

QL conceived and designed the experiments. ZG performed the experiments. QL and QT analyzed data. YM contributed to the preparation of reagents/materials/analysis tools. QL wrote the manuscript. SC supervised this study. All authors contributed to the article and approved the submitted version.

## Conflict of Interest

The authors declare that the research was conducted in the absence of any commercial or financial relationships that could be construed as a potential conflict of interest.

## Publisher’s Note

All claims expressed in this article are solely those of the authors and do not necessarily represent those of their affiliated organizations, or those of the publisher, the editors and the reviewers. Any product that may be evaluated in this article, or claim that may be made by its manufacturer, is not guaranteed or endorsed by the publisher.

## References

[1] Sharifi E Bigham A Yousefiasl S Trovato M Ghomi M Esmaeili Y. Mesoporous Bioactive Glasses in Cancer Diagnosis and Therapy: Stimuli-Responsive, Toxicity, Immunogenicity, and Clinical Translation. Adv Sci (Weinh) (2022) 9(2):e2102678. doi: 10.1002/advs.202102678 34796680 PMC8805580

[2] Kuroki L Guntupalli SR. Treatment of Epithelial Ovarian Cancer. BMJ (2020) 371:m3773. doi: 10.1136/bmj.m3773 33168565

[3] Saika K Sobue T. Cancer Statistics in the World. Gan To Kagaku Ryoho (2013) 40(13):2475–80. 24335357

[4] Agraharam G Girigoswami A Girigoswami K. Myricetin: A Multifunctional Flavonol in Biomedicine. Curr Pharmacol Rep (2022) 8(1):48–61. doi: 10.1007/s40495-021-00269-2 35036292 PMC8743163

[5] Coêlho CFF Souza ILS Chagas VT Ribeiro NLX Pinto BAS França LM Paes AMA. Myricetin Improves Metabolic Outcomes But Not Cognitive Deficit Associated to Metabolic Syndrome in Male Mice. Food Funct (2021) 12(8):3586–96. doi: 10.1039/D1FO00073J 33900338

[6] Imran M Saeed F Hussain G Imran A Mehmood Z Gondal TA. Myricetin: A Comprehensive Review on its Biological Potentials. Food Sci Nutr (2021) 9(10):5854–68. doi: 10.1002/fsn3.2513 PMC8498061 34646551

[7] Xu Y Wang S Chan HF Lu H Lin Z He C. Dihydromyricetin Induces Apoptosis and Reverses Drug Resistance in Ovarian Cancer Cells by P53-Mediated Downregulation of Survivin. Sci Rep (2017) 7(1):46060. doi: 10.1038/srep46060 28436480 PMC5402300

[8] Zhang Q Liu J Liu B Xia J Chen N Chen X. Dihydromyricetin Promotes Hepatocellular Carcinoma Regression *via* a P53 Activation-Dependent Mechanism. Sci Rep (2014) 4(1):4628. doi: 10.1038/srep04628 24717393 PMC3982169

[9] Crocetto F di Zazzo E Buonerba C Aveta A Pandolfo SD Barone B. Kaempferol, Myricetin and Fisetin in Prostate and Bladder Cancer: A Systematic Review of the Literature. Nutrients (2021) 13(11):3750. doi: 10.3390/nu13113750 34836005 PMC8621729

[10] Kao SJ Lee WJ Chang JH Chow JM Chung CL Hung WY. Suppression of Reactive Oxygen Species-Mediated ERK and JNK Activation Sensitizes Dihydromyricetin-Induced Mitochondrial Apoptosis in Human Nonsmall Cell Lung Cancer. Environ Toxicol (2017) 32(4):1426–38. doi: 10.1002/tox.22336 27539140

[11] Delfi M Sartorius R Ashrafizadeh M Sharifi E Zhang Y De Berardinis P. Self-Assembled Peptide and Protein Nanostructures for Anti-Cancer Therapy: Targeted Delivery, Stimuli-Responsive Devices and Immunotherapy. Nano Today (2021) 38:101119. doi: 10.1016/j.nantod.2021.101119 34267794 PMC8276870

[12] Martínez-Limón A Joaquin M Caballero M Posas F de Nadal E. The P38 Pathway: From Biology to Cancer Therapy. Int J Mol Sci (2020) 21(6):1913. doi: 10.3390/ijms21061913 PMC7139330 32168915

[13] Banno K Yanokura M Iida M Adachi M Nakamura K Nogami Y. Application of MicroRNA in Diagnosis and Treatment of Ovarian Cancer. BioMed Res Int (2014) 2014:232817. doi: 10.1155/2014/232817 24822185 PMC4009316

[14] Mourouti N Kontogianni MD Papavagelis C Panagiotakos DB. Diet and Breast Cancer: A Systematic Review. Int J Food Sci Nutr (2015) 66:1–42. doi: 10.3109/09637486.2014.950207 25198160

[15] Crocetto F di Zazzo E Buonerba C Aveta A Pandolfo SD Barone B. A Review on Myricetin as a Potential Therapeutic Candidate for Cancer Prevention. 3 Biotech (2020) 10(5):211. doi: 10.1007/s13205-020-02207-3 PMC7181463 32351869

[16] Soleimani M Sajedi N. Myricetin Apoptotic Effects on T47D Breast Cancer Cells is a P53-Independent Approach. Asian Pac J Cancer Prev (2020) 21(12):3697–704. doi: 10.31557/APJCP.2020.21.12.3697 PMC8046314 33369470

[17] Mondal S Jana J Sengupta P Jana S Chatterjee S. Myricetin Arrests Human Telomeric G-Quadruplex Structure: A New Mechanistic Approach as an Anticancer Agent. Mol Biosyst (2016) 12(8):2506–18. doi: 10.1039/C6MB00218H 27249025

[18] Bitew M Desalegn T Demissie TB Belayneh A Endale M Eswaramoorthy R. Pharmacokinetics and Drug-Likeness of Antidiabetic Flavonoids: Molecular Docking and DFT Study. PloS One (2021) 16(12):e0260853. doi: 10.1371/journal.pone.0260853 34890431 PMC8664201

[19] Kang HR Moon JY Ediriweera MK Song YW Cho M Kasiviswanathan D. Dietary Flavonoid Myricetin Inhibits Invasion and Migration of Radioresistant Lung Cancer Cells (A549-IR) by Suppressing MMP-2 and MMP-9 Expressions Through Inhibition of the FAK-ERK Signaling Pathway. Food Sci Nutr (2020) 8(4):2059–67. doi: 10.1002/fsn3.1495 PMC7174229 32328272

[20] Tavsan Z Kayali HA. Flavonoids Showed Anticancer Effects on the Ovarian Cancer Cells: Involvement of Reactive Oxygen Species, Apoptosis, Cell Cycle and Invasion. BioMed Pharmacother (2019) 116:109004. doi: 10.1016/j.biopha.2019.109004 31128404

[21] Oh JY Giles N Landar A Darley-Usmar V. Accumulation of 15-Deoxydelta(12,14)-Prostaglandin J2 Adduct Formation With Keap1 Over Time: Effects on Potency for Intracellular Antioxidant Defence Induction. Biochem J (2008) 411(2):297–306. doi: 10.1042/BJ20071189 18237271 PMC2683789

[22] Kovacic P Jacintho JD. Mechanisms of Carcinogenesis: Focus on Oxidative Stress and Electron Transfer. Curr Med Chem (2001) 8(7):773–96. doi: 10.2174/0929867013373084 11375749

[23] Block KI Koch AC Mead MN Tothy PK Newman RA Gyllenhaal C. Impact of Antioxidant Supplementation on Chemotherapeutic Toxicity: A Systematic Review of the Evidence From Randomized Controlled Trials. Int J Cancer (2008) 123(6):1227–39. doi: 10.1002/ijc.23754 18623084

[24] Hsu YJ Ho CS Lee MC Ho CS Huang CC Kan NW. Protective Effects of Resveratrol Supplementation on Contusion Induced Muscle Injury. Int J Med Sci (2020) 17(1):53–62. doi: 10.7150/ijms.35977 31929738 PMC6945554

[25] Xie J Chen MH Ying CP Chen MY. Neferine Induces P38 MAPK/JNK1/2 Activation to Modulate Melanoma Proliferation, Apoptosis, and Oxidative Stress. Ann Transl Med (2020) 8(24):1643. doi: 10.21037/atm-20-7201 33490155 PMC7812205

[26] Wiemer EA. Stressed Tumor Cell, Chemosensitized Cancer. Nat Med (2011) 17(12):1552–4. doi: 10.1038/nm.2593 22146456

[27] Luo Y Zou P Zou J Wang J Zhou D Liu L. Autophagy Regulates ROS-Induced Cellular Senescence *via* P21 in a P38 Mapkα Dependent Manner. Exp Gerontol (2011) 46(11):860–7. doi: 10.1016/j.exger.2011.07.005 PMC3390188 21816217

[28] Radisky DC Levy DD Littlepage LE Liu H Nelson CM Fata JE. Rac1b and Reactive Oxygen Species Mediate MMP-3-Induced EMT and Genomic Instability. Nature (2005) 436(7047):123–7. doi: 10.1038/nature03688 PMC2784913 16001073

