# Supplementary figures and images for: Myricetin Suppresses Ovarian Cancer In Vitro by Activating the p38/Sapla Signaling Pathway and Suppressing Intracellular Oxidative Stress
Source: Front Oncol. 2022 May 11;12:903394. doi: 10.3389/fonc.2022.903394 (PMC9130763; doi:10.3389/fonc.2022.903394)

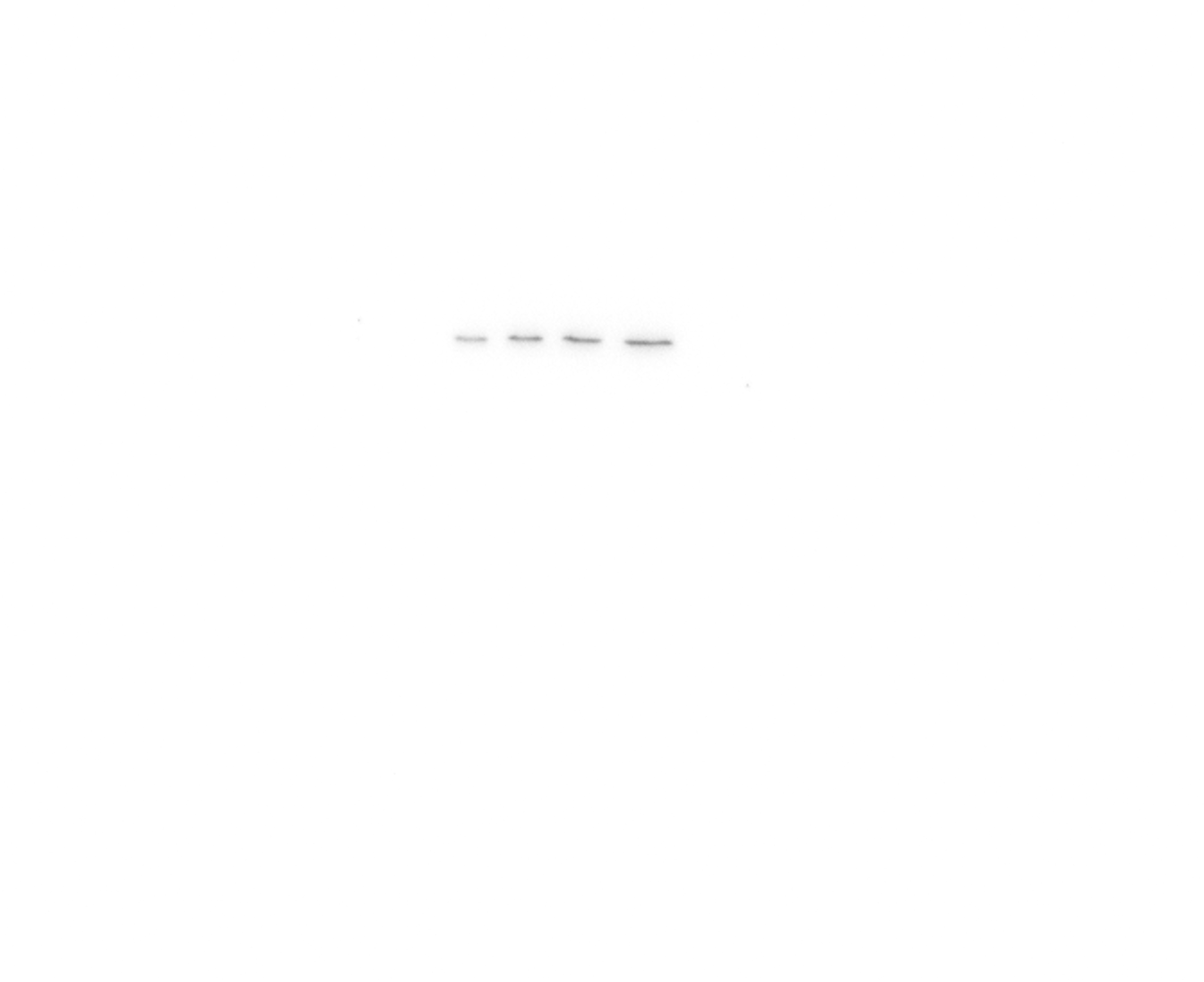

Supplement: Supplementary file 1 [file DataSheet_1.zip › FIG6/11 c-c3.tif]

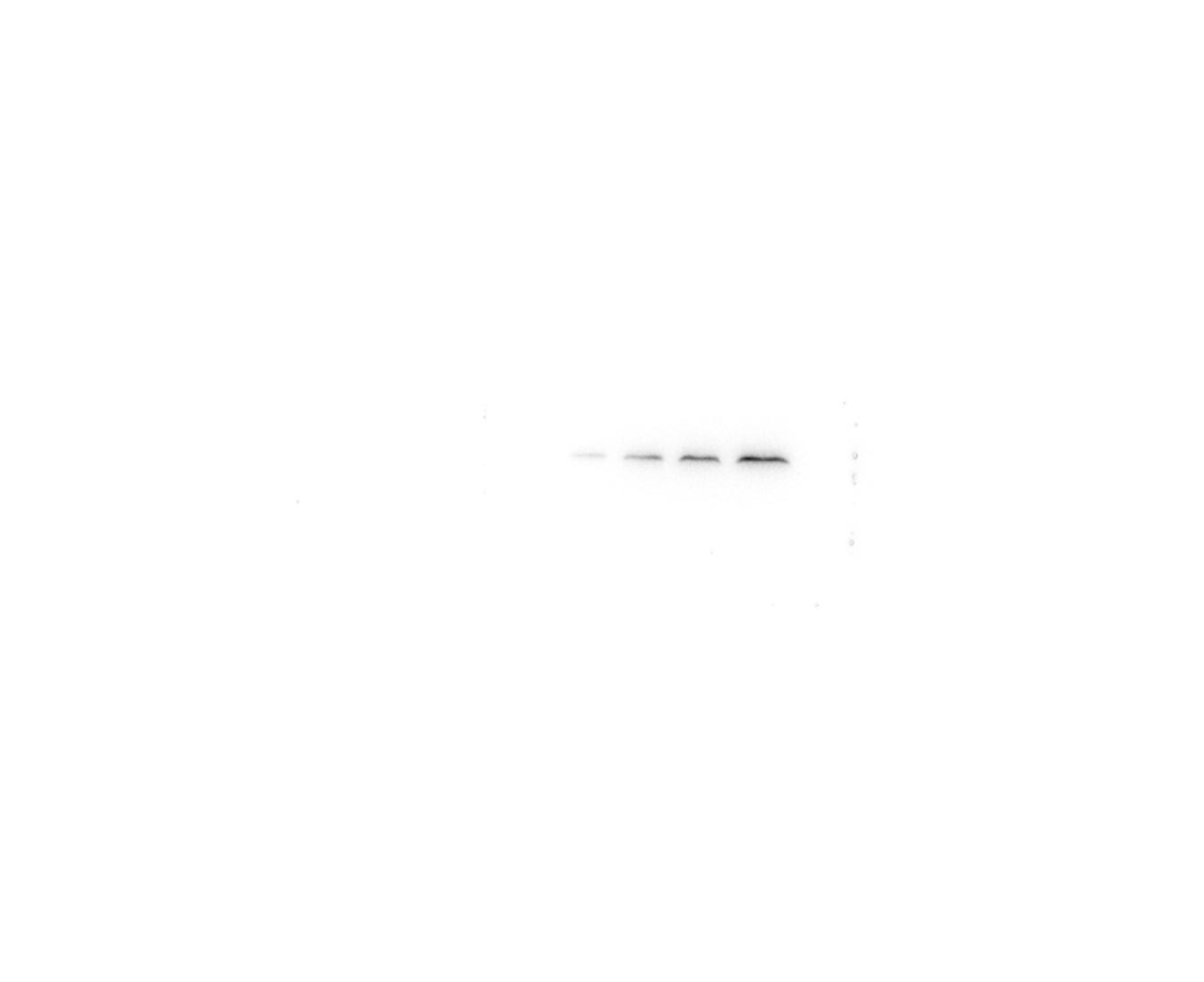

Supplement: Supplementary file 1 [file DataSheet_1.zip › FIG6/12 c-c-9.tif]

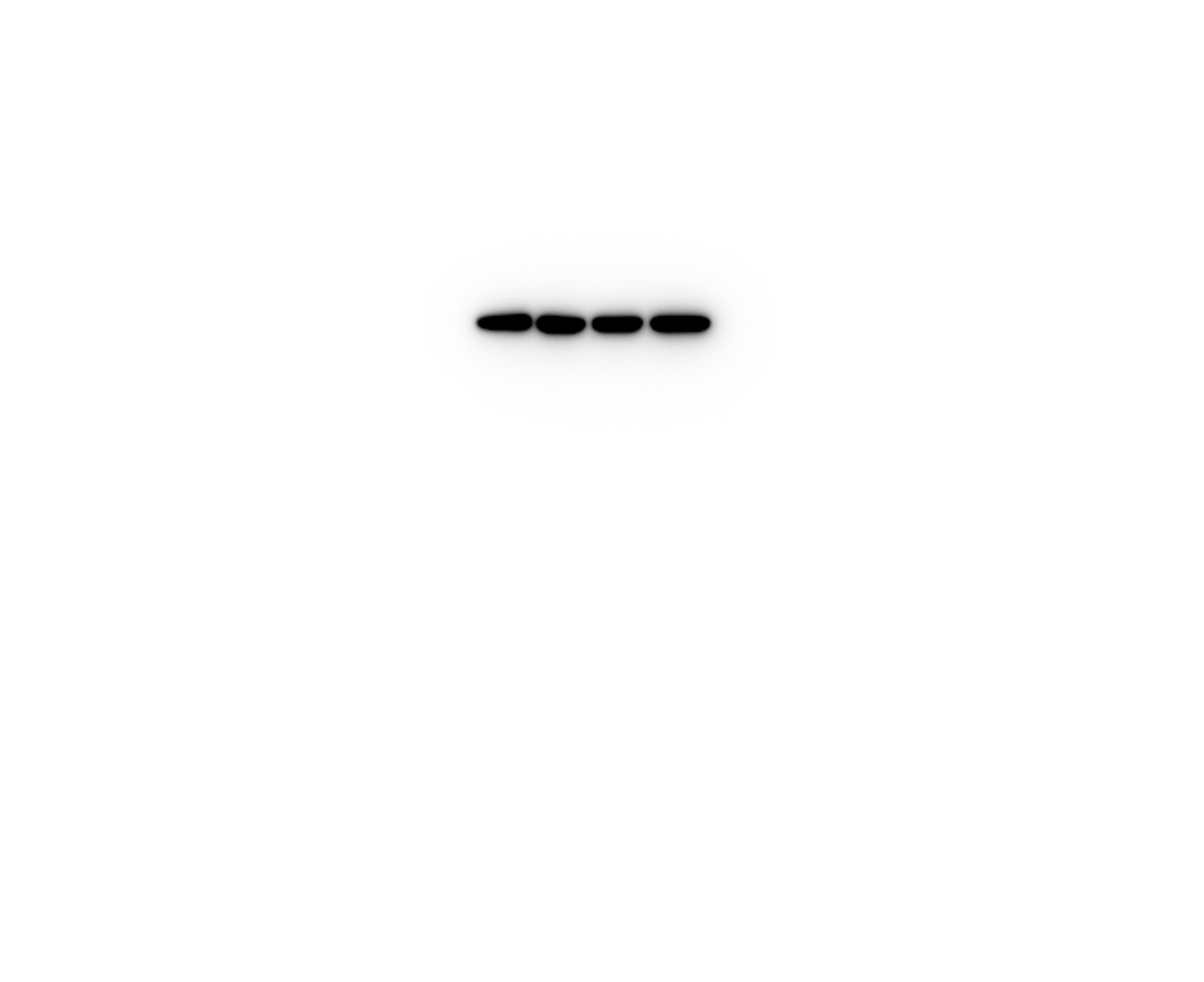

Supplement: Supplementary file 1 [file DataSheet_1.zip › FIG6/13 GAPDH.tif]

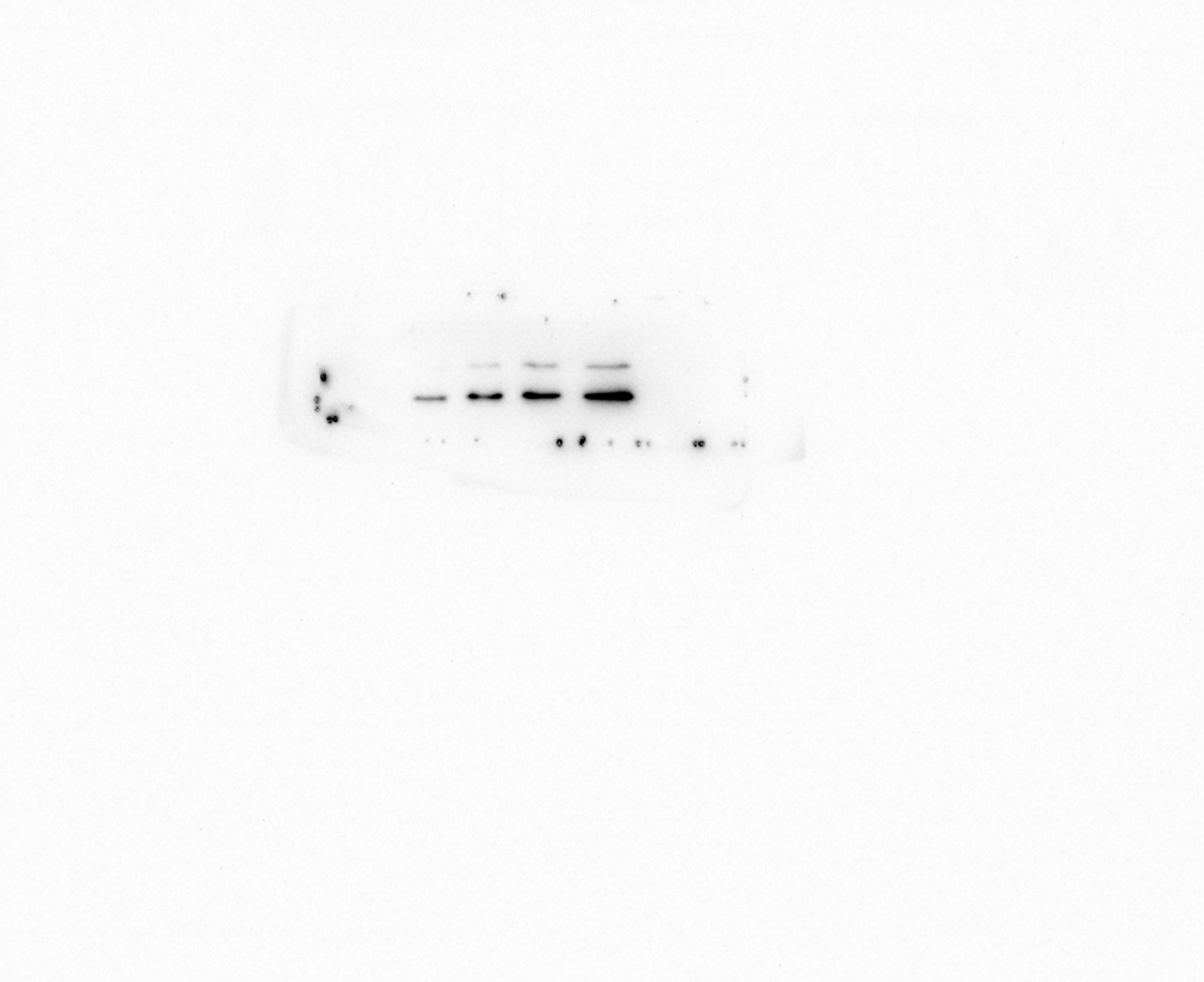

Supplement: Supplementary file 1 [file DataSheet_1.zip › FIG6/2 p-p38.tif]

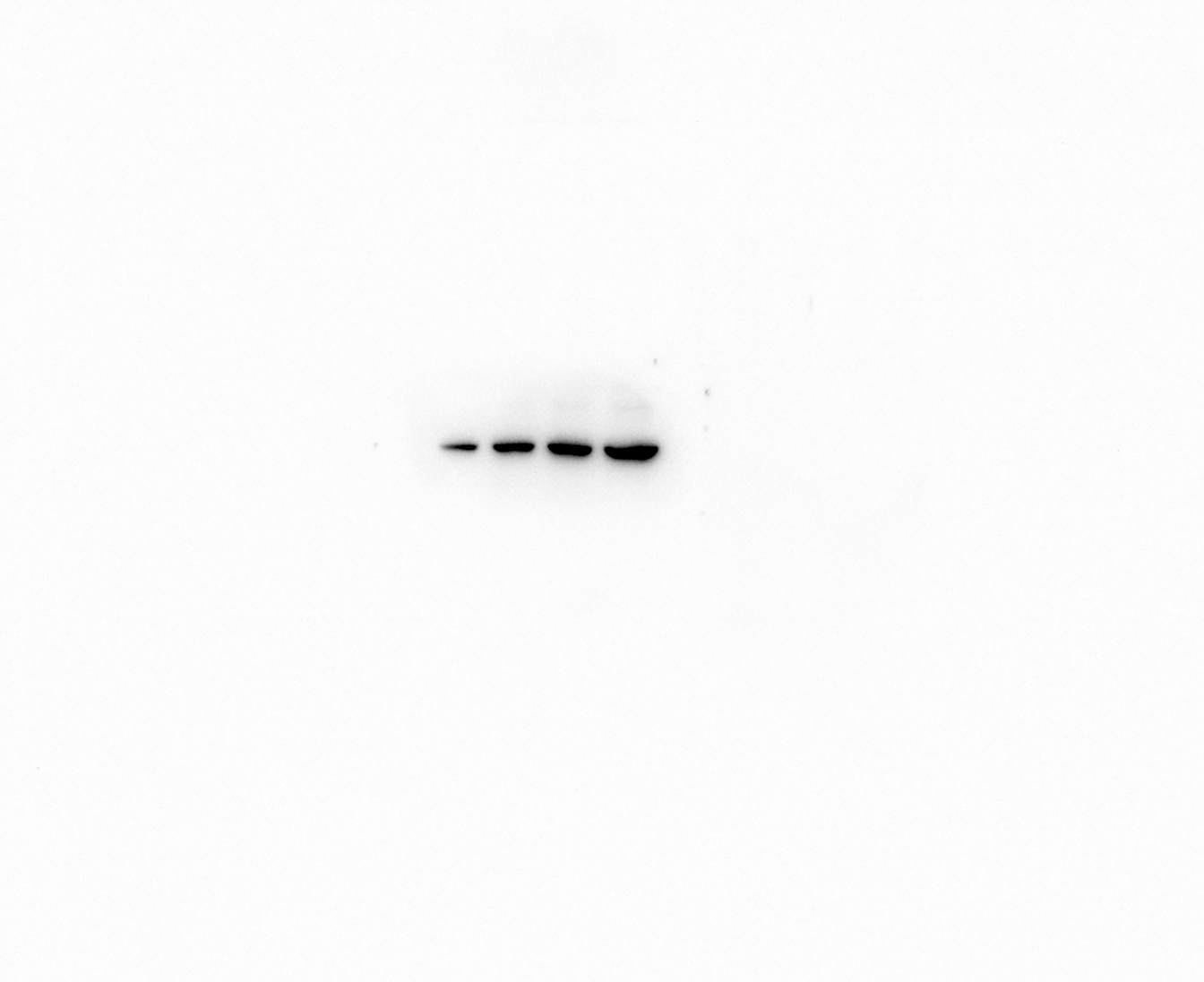

Supplement: Supplementary file 1 [file DataSheet_1.zip › FIG6/3 Sapla.tif]

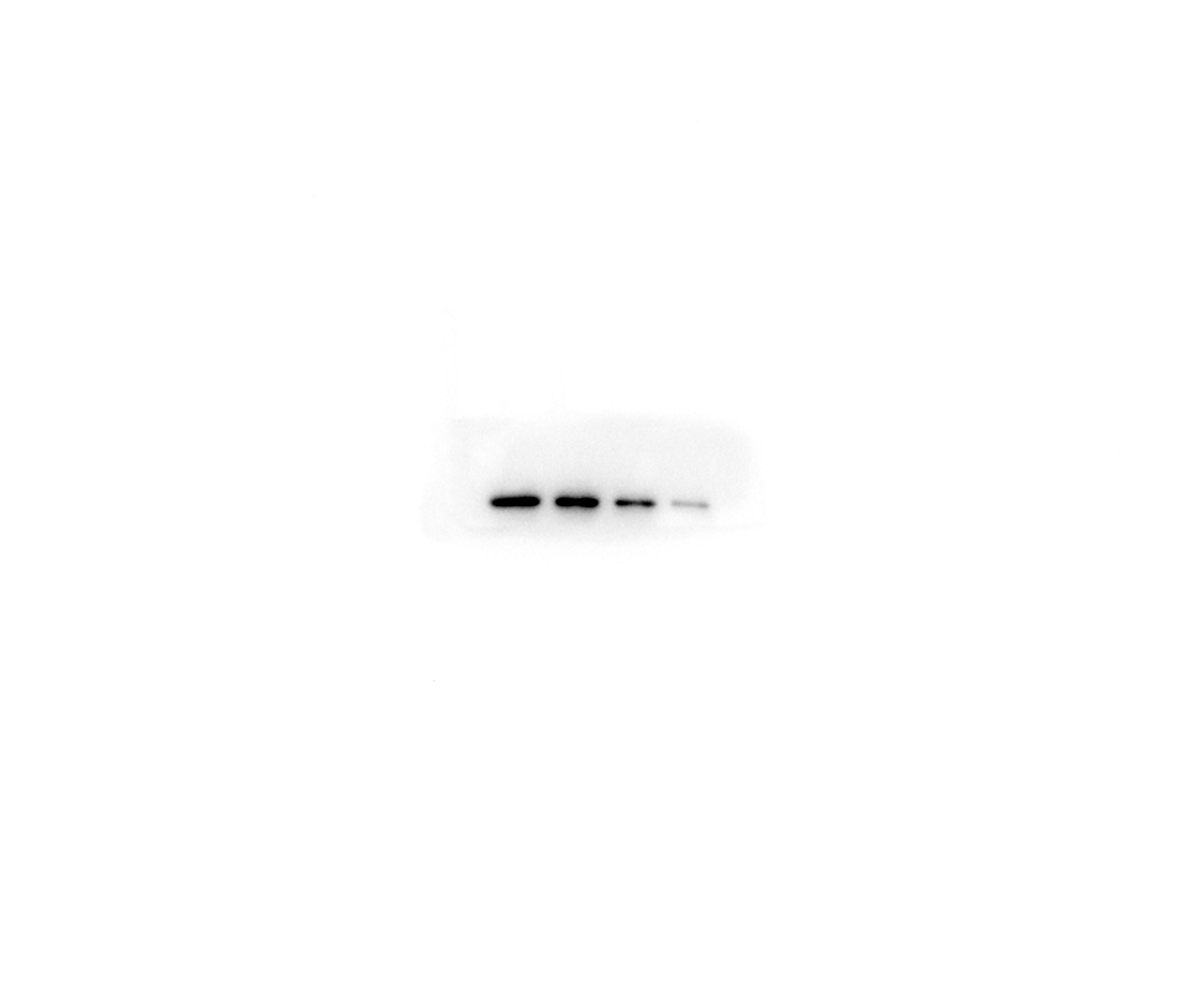

Supplement: Supplementary file 1 [file DataSheet_1.zip › FIG6/4 EGFR.tif]

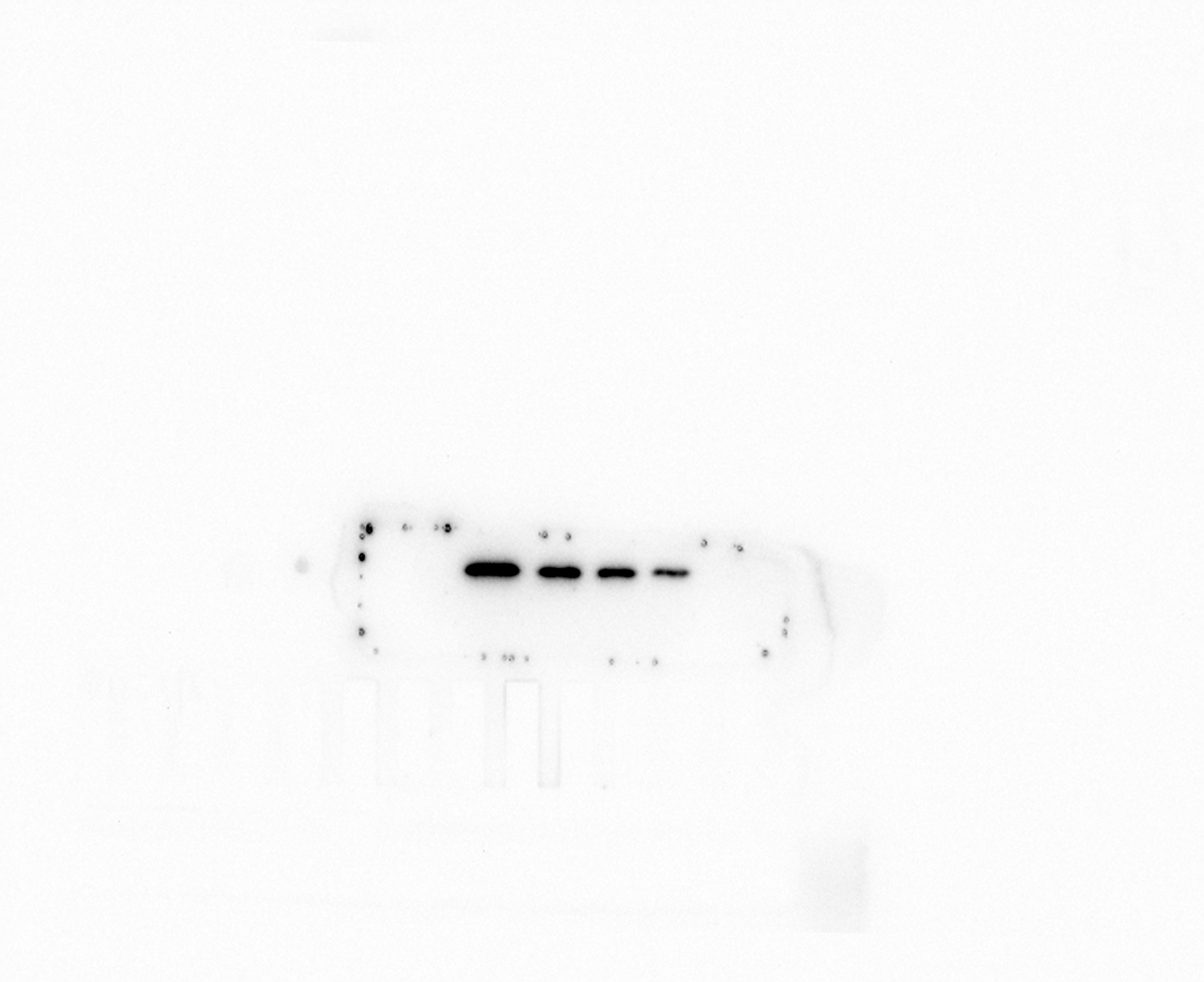

Supplement: Supplementary file 1 [file DataSheet_1.zip › FIG6/5 MMP-2.tif]

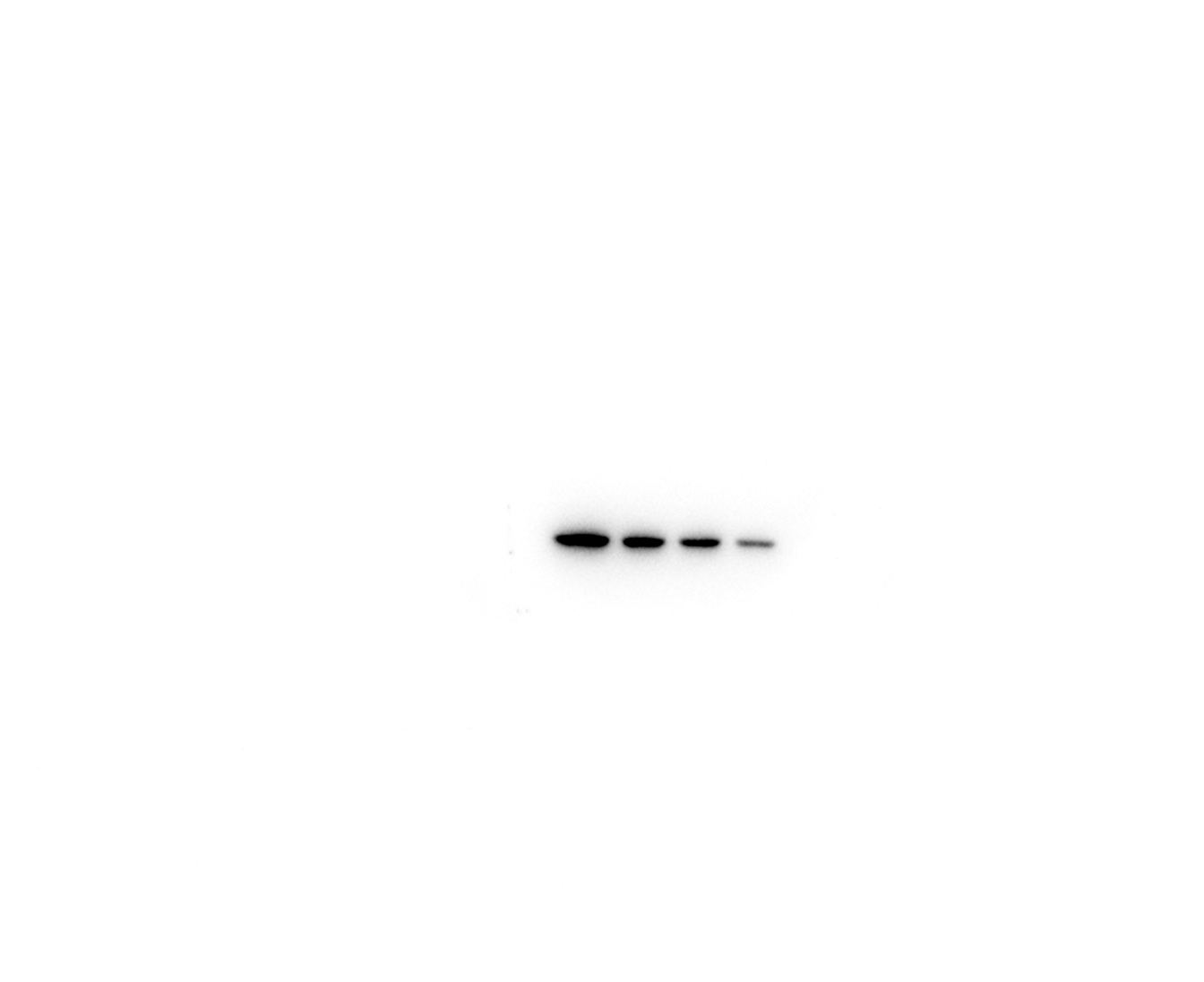

Supplement: Supplementary file 1 [file DataSheet_1.zip › FIG6/6 MMP-3.tif]

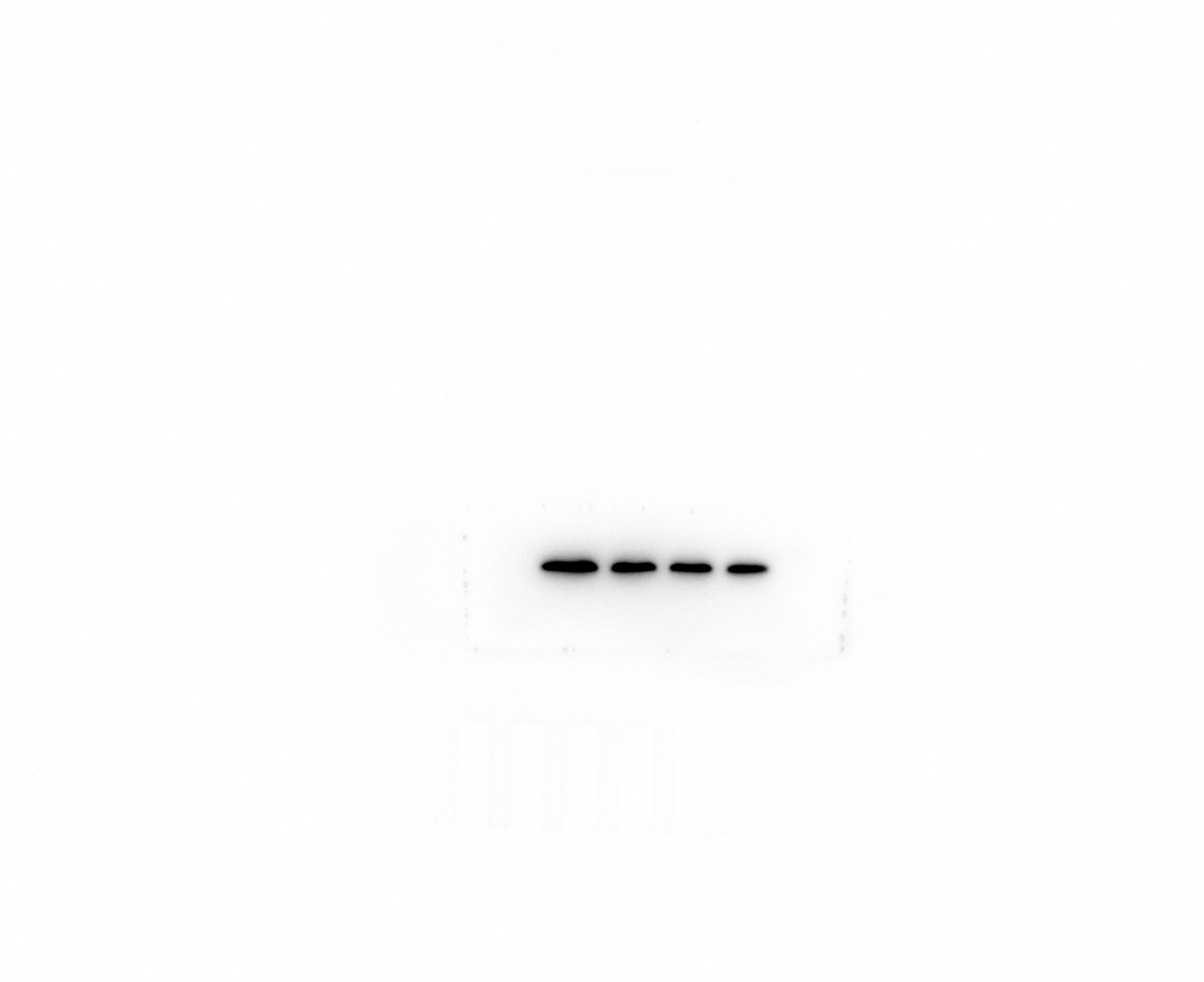

Supplement: Supplementary file 1 [file DataSheet_1.zip › FIG6/7 MMP-9.tif]

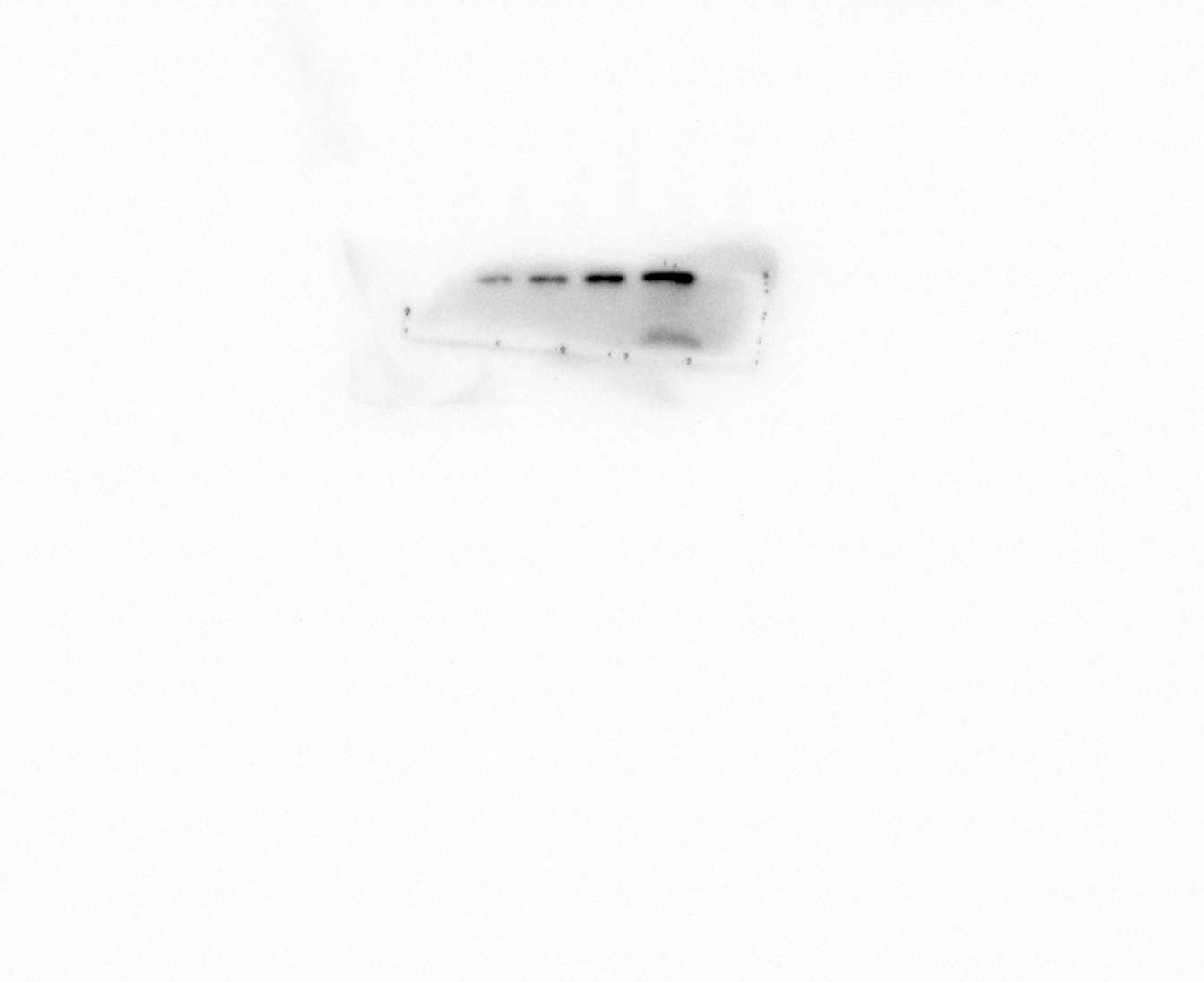

Supplement: Supplementary file 1 [file DataSheet_1.zip › FIG6/8 Bax.tif]

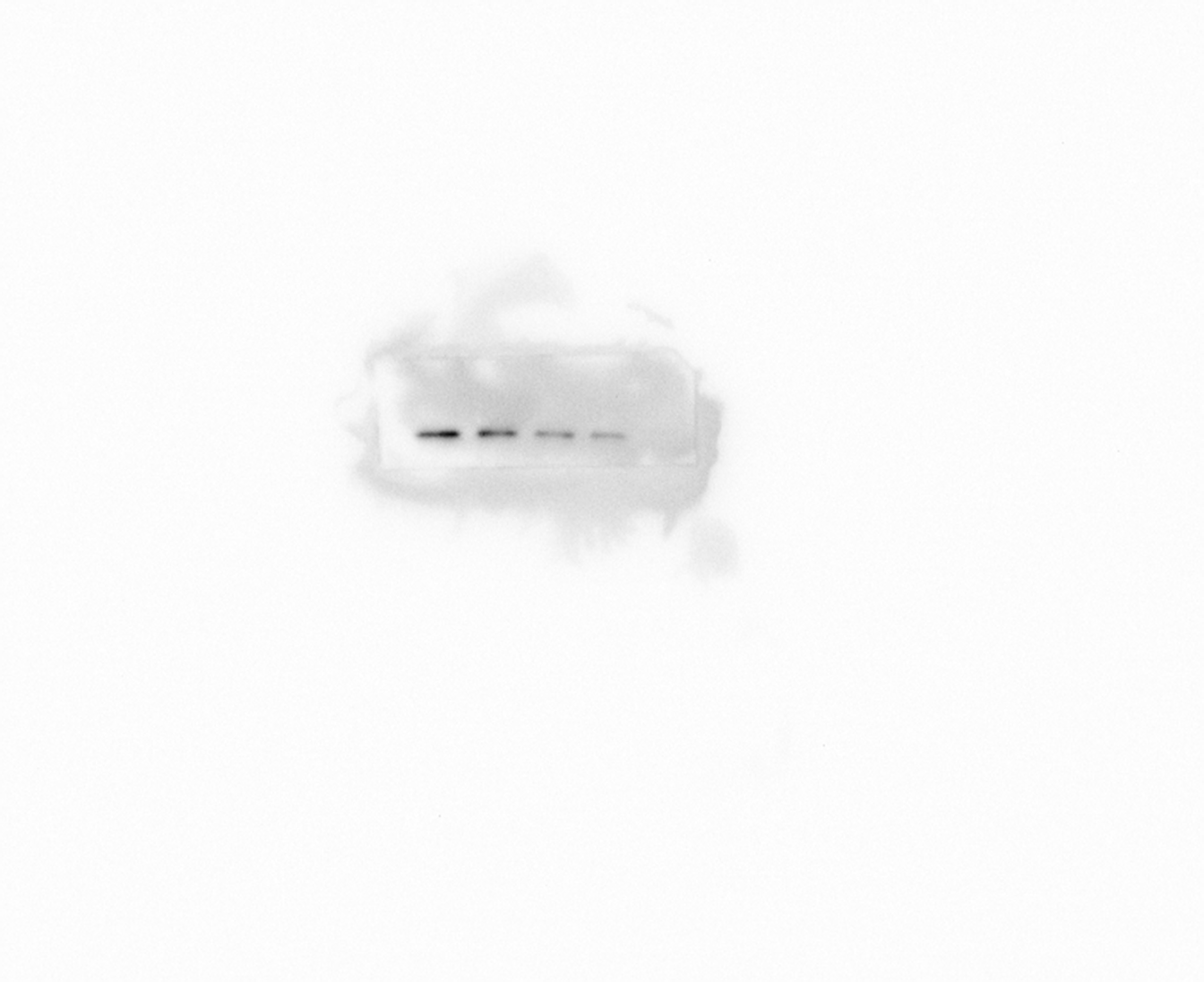

Supplement: Supplementary file 1 [file DataSheet_1.zip › FIG6/9 Bcl-2.tif]
